# Effectiveness of spinal cord stimulation for painful camptocormia with Pisa syndrome in Parkinson’s disease: a case report

**DOI:** 10.1186/s12883-017-0926-y

**Published:** 2017-08-03

**Authors:** Hisanao Akiyama, Saki Nukui, Masashi Akamatu, Yasuhiro Hasegawa, Osamu Nishikido, Soichiro Inoue

**Affiliations:** 1 Department of Neurology, St. Marianna University School of Medicine, 2-16-1 Sugao, Miyamae-ku, Kawasaki, Kanagawa 216-8511 Japan; 2 Department of Anesthesiology, St. Marianna University School of Medicine, Kawasaki, Kanagawa Japan; 3 Department of Palliative Medicine, Showa University Northern Yokohama Hospital, Yokohama, Kanagawa Japan

**Keywords:** Spinal cord stimulation, Parkinson’s disease, Painful truncal postural abnormality, Camptocormia, Pisa syndrome

## Abstract

**Background:**

Spinal cord stimulation (SCS) has recently been reported to be effective for truncal postural abnormalities such as camptocormia and Pisa syndrome in Parkinson’s disease. In this case report, we describe a case of a woman with Parkinson’s disease in whom SCS was effective for painful camptocormia with Pisa syndrome.

**Case presentation:**

A 65-year-old woman was admitted to our hospital because of painful camptocormia. She had noticed resting tremor in the left upper limb and aprosody at 48 years of age. She was diagnosed as having Parkinson’s disease (Hoehn & Yahr stage 1) at 53 years of age. Cabergoline was started during that same year, with subsequent addition of selegiline hydrochloride; the symptoms of parkinsonism disappeared. Wearing-off occurred when she was 57 years old, 3 years after starting carbidopa/levodopa, and truncal postural abnormalities—painful camptocormia with Pisa syndrome to the right—appeared at 58 years of age. These symptoms worsened despite adjustment of her oral medications, and deep brain stimulation (DBS) was performed when she was 60 years old. The truncal postural abnormalities improved after DBS, and she could travel abroad at 61 years of age. However, from 62 years of age, painful camptocormia with Pisa syndrome to the right reappeared. The pain was unsuccessfully treated with oral analgesics, radiofrequency coagulation of the dorsal and medial branches of the lumbar spinal nerve, and lumbar epidural block. Finally, SCS was performed for the pain relief. The pain disappeared immediately after SCS and her posture then gradually improved. Unified Parkinson’s Disease Rating Scale score improved from 48 to 34 points and Timed Up and Go Test improved from 15 s to 7 s after SCS.

**Conclusions:**

This case suggests that SCS may be effective for improving painful truncal postural abnormalities and motor complications of Parkinson’s disease. Pain relief or a direct effect on the central nervous system by SCS was considered to explain the alleviation of these symptoms.

## Background

Truncal postural abnormalities such as camptocormia and Pisa syndrome, as well as Parkinson’s-related and -unrelated pain, are not uncommon in patients with Parkinson’s disease [1, 2], and medical treatment for these symptoms is often difficult. An alternative treatment strategy is spinal cord stimulation (SCS), which was recently reported to be effective for truncal postural abnormalities, chronic pain, and motor symptoms of Parkinson’s disease, though the mechanism of SCS remains unclear [3–5].

In this case report, we present a case of painful camptocormia that was effectively treated with SCS in a woman with Parkinson’s disease. In addition, we briefly review the past literature.

## Case presentation

A 65-year-old woman with Parkinson’s disease was admitted to our hospital because of painful camptocormia. She had a past medical history of postoperative adhesive intestinal obstruction after surgery for cancer of the uterine body. She was a housewife and had no significant social history of drinking and smoking. She had noticed resting tremor of the left upper limb and aprosody at 48 years of age. When she was 53 years old, she was diagnosed as having Parkinson’s disease (Hoehn & Yahr stage 1) according to the UK Parkinson’s Disease Society Brain Bank criteria [6]. At the time of diagnosis, magnetic resonance imaging (MRI) of the head showed no abnormality such as bilateral outer layer slit high-intensity lesions on T2-weighted imaging (suggesting putaminal atrophy), hot cross bun sign, and brain stem and cerebellar atrophy. Oral administration of cabergoline and selegiline hydrochloride resulted in improvement in her parkinsonism symptoms, but their effectiveness decreased gradually after 6 months. Subsequently, carbidopa/levodopa 100 mg was started at 54 years of age, and the wearing-off phenomenon occurred when she was 57 years old. Painful camptocormia with Pisa syndrome to the right appeared at the age of 58 years. Despite several adjustments to her medications, such as increasing up to the maximum dose of 400 mg carbidopa/levodopa or decreasing the dose of dopamine agonist, her symptoms worsened. She underwent deep brain stimulation (DBS) at 60 years of age. Model 3387 DBS leads (Medtronic, Inc., Minneapolis, MN; medical device certificate number 20700BZY00880000) were inserted into both subthalamic nuclei and connected to two neurostimulators (Activa SC; Medtronic, Inc., Minneapolis, MN; medical device certificate number 22300BZX00414000). The DBS parameters were as follows. Right internal generator program: anode, contact case; cathode, contacts Nos. 2 and 3; amplitude, 3.5 V; pulse width, 60 μs; frequency, 60 Hz. Left internal generator program: anode, contacts case; cathode, contact Nos. 2 and 3; amplitude, 3.2 V; pulse width, 60 μs; frequency, 60 Hz maximally. By 1 year after commencing DBS, when she was 61 years old, the camptocormia had disappeared completely. As a result, she could travel abroad independently. However, the painful camptocormia with Pisa syndrome reappeared at 62 years of age. Oral administration of analgesics, radiofrequency coagulation of the medial and dorsal branches of the spinal nerves L2/3, 3/4, and 4/5, and lumbar epidural block were administered for the lumbar pain, but were ineffective. At that time, ^123^I–ioflupane dopamine transporter SPECT (DAT scan) revealed severe hypo-accumulation (right dominant) in both striata; and head CT revealed no brain stem and cerebellar atrophy at 64 years of age. She was hospitalized for SCS in March 2016 because a temporary 1-week testing trial of SCS utilizing a pair of Vectris SureScan^®^ MRI 1 × 8 Compact leads (Medtronic, Inc.; medical device certificate number 22500BZX00346000) and external generator External Stim^®^ (Medtronic, Inc.; medical device certificate number 22100BZX00196000) in February was found to be effective.

**Fig. 1:**
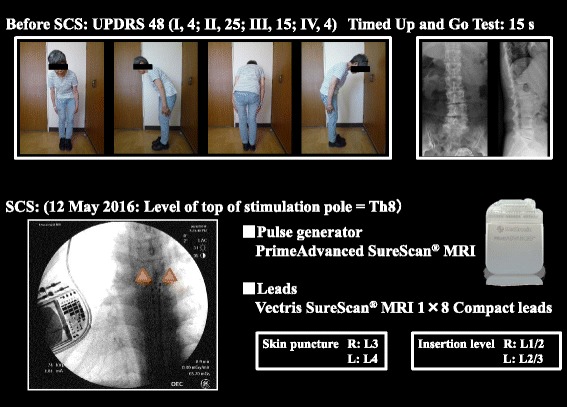
Painful camptocormia and SCS device. The patient’s camptocormia with Pisa syndrome to the right (top *left*). Spinal X-ray revealed mild degenerative lumbar spondylosis (top *right*). For SCS, a PrimeAdvanced SureScan^®^ MRI pulse generator was implanted and a pair of Vectris SureScan^®^ MRI 1 × 8 Compact leads were inserted percutaneously

On admission, her oral medications were carbidopa/levodopa 800 mg in 4 divided doses (2–2–3–1 tablets), pramipexole 4.5 mg in 3 divided doses, istradefylline 20 mg once daily, midodrine hydrochloride 8 mg in 2 divided doses, limaprost alfadex 15 μg in 3 divided doses, teprenone 150 mg in 3 divided doses, and loxoprofen 180 mg in 3 divided doses. General examination revealed no abnormalities except for the bilateral anterior thoracic DBS generators. On neurological examination she was fully conscious, with aprosody, and masked facies. The cranial nerves, motor system, reflexes, sensory system, coordination, and meningeal signs were all normal, though urinary incontinence was present. Regarding extrapyramidal symptoms, there was no resting tremor or rigidity in the limbs, but bradykinesia, postural instability, and painful camptocormia with Pisa syndrome to the right were observed (Fig. 1, top left). Urinalysis, blood tests, electrocardiogram, and chest X-ray revealed no abnormalities except for mild degenerative lumbar spondylosis on spinal X-ray (Fig. 1, top right). Cardiac meta-iodobenzylguanidine scintigraphy and DAT scan were not performed during this admission. The Unified Parkinson’s Disease Rating Scale (UPDRS) score was 48 (part I, 4; II, 25; III, 15; IV, 4) and Timed Up and Go Test was 15 s before SCS implantation.

After admission, on 12 May 2016, a PrimeAdvanced SureScan^®^ MRI pulse generator (Medtronic, Inc.; Medical device certificate number 22500BZX00345000) was implanted and a pair of Vectris SureScan^®^ MRI 1 × 8 Compact leads were inserted percutaneously. Skin was punctured at the level of L3 on the right and L4 on the left, and the top of the stimulation points was at the level of Th8 (Fig. 1, bottom) as a result of adjustment to achieve pain relief with mild paresthesia. For the SCS implant, the pulse generator was placed in the subcutaneous pocket of the lower abdomen. SCS parameters were as follows. Program 1: anode, contacts Nos. 2 and 10; cathode, contact Nos. 0, 1, and 8; amplitude, 2.5 V; pulse width, 450 μs; and frequency, 7 Hz. Program 2: anode, contact No. 0; cathode, contact No. 1; amplitude, 3.5 V; pulse width, 250 μs and frequency, 7 Hz.

**Fig. 2:**
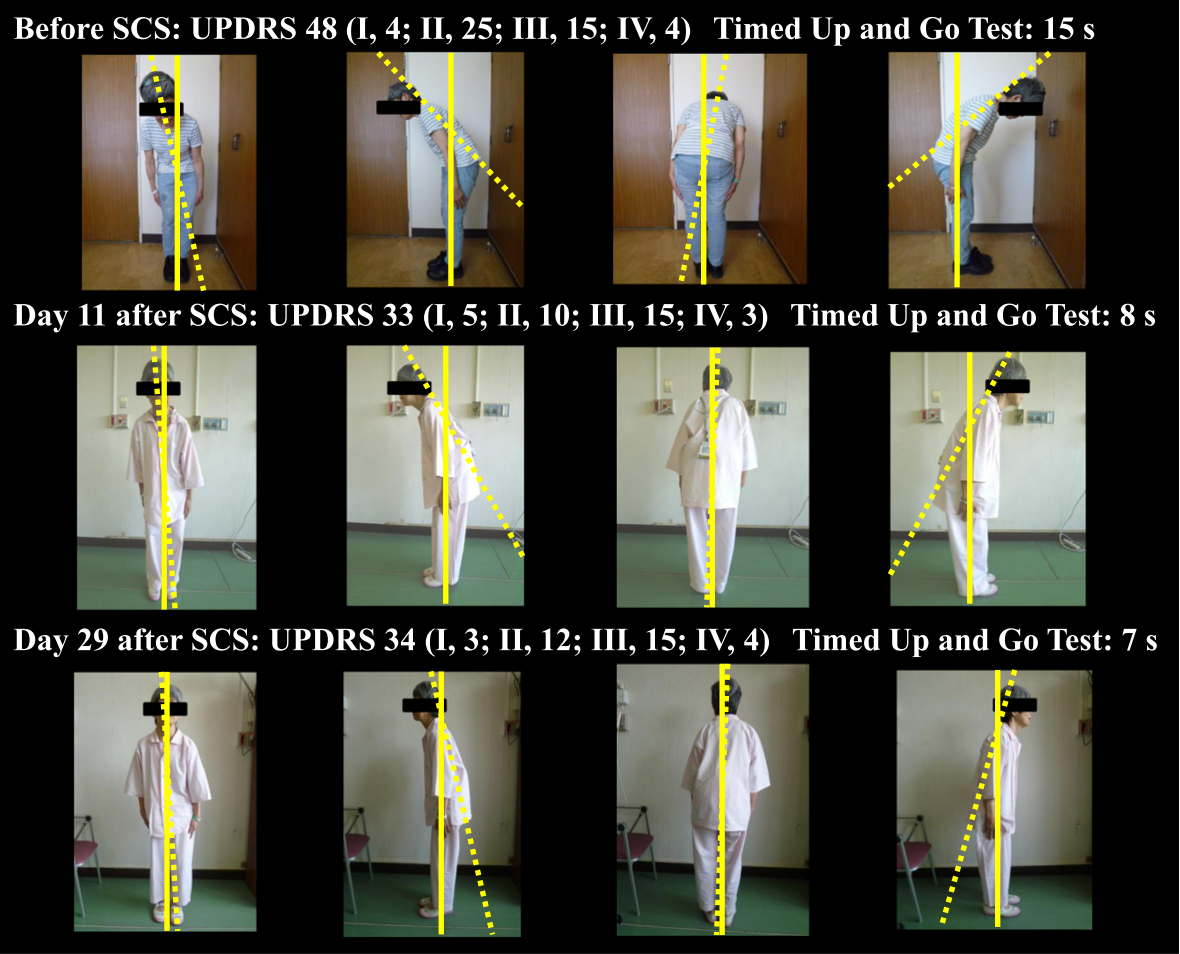
Improvement of painful camptocormia with Pisa syndrome after SCS. Painful camptocormia and Pisa syndrome to the right improved gradually after SCS

Pain was markedly improved after SCS implantation, with the visual analog scale score improving from 10 points before SCS to 2 points after SCS on the day of surgery. The camptocormia also improved gradually after SCS **(**Fig. 2), UPDRS score improved from 48 points (part II, 25) to 33 points (part I, 5; II, 10; III, 15; IV, 3), with a particularly large improvement for part II (which evaluates activities of daily living). The Timed Up and Go Test markedly improved from 15 s before SCS to 8 s on the 11th day after SCS at the same time of day (always measured at 14:00, during the ON period). By the 29th day after SCS, the camptocormia had further improved; the UPDRS score was 34 points (part II, 12), and the Timed Up and Go Test was 7 s during the ON period. In addition, the following improvements were observed in scores for chronic pain, psychiatric symptoms, and disability after SCS: Numerical Rating Scale, 9 to 7; Leeds Assessment of Neuropathic Symptoms and Signs Pain Scale, 11 to 5; Hospital Anxiety and Depression Scale 4 to 9; EuroQol (EQ-5S), 0.101 to 0.208; and Pain Disability Assessment Scale, 39 to 36. Her general condition has remained satisfactory for 6 months as of this writing.

## Discussion

Postural abnormalities such as camptocormia occur with a prevalence of approximately 3.0–12.7% in patients with Parkinson’s disease [1]. Camptocormia is one of the motor symptoms that adversely affect activities of daily living. Many possible causes of camptocormia have been reported, including muscle rigidity and dystonia in the abdomen, proprioceptive deficits, adverse effects of dopamine agonists, soft tissue imbalance, and fatty degeneration and myopathy in the back muscles, but details remain unclear [7]. Although there is no effective curative therapy, several symptomatic therapies are reported to be effective, including dosage of oral carbidopa/levodopa or dopamine agonists, botulinum toxin injections, and DBS [3, 8–11]. Meanwhile, pain in the low back and legs is common in Parkinson’s disease, with a prevalence of approximately 40–85% [3]. Camptocormia occurs with or without pain, and either way, it is an important factor impairing activities of daily living. However, the mechanism underlying the onset of pain is still not known [2]. Pain can be Parkinson’s disease-related or -unrelated, but strictly distinguishing between the two types is clinically difficult. Given that drug resistance commonly occurs, SCS has been investigated, which relieves pain by electrically stimulating the posterior funiculus. SCS was recently shown to be particularly effective in patients with Parkinson’s disease-unrelated pain and/or postural abnormalities [3–5]. In the present case, SCS therapy was effective for a patient with Parkinson’s disease who had camptocormia accompanied by intractable chronic pain in the absence of bone lesions and who had no history of botulinum toxin injection, showed resistance to oral carbidopa/levodopa and dopamine agonists, and had previously experienced transient alleviation of symptoms by DBS.

The mechanism of pain relief by SCS is generally explained by the gate control theory proposed by Melzack and Wall in 1965, but the details remain unclear [12]. Based on the gate control therapy, several mechanisms involved in pain relief by SCS have been proposed. For instance, stimulation of the posterior funiculus by SCS causes excitation of thick Aβ sensory fibers and excitation of thick Aβ fibers leads to suppression of sensory pain C fibers at the spinal dorsal horn. Blockage of pain at the spinal dorsal horn and the spinothalamic tract occurs by retrograde impulse conduction, which activates inhibitory neurons, increases GABA release, and reduces excitatory glutamic acid level. Other mechanisms have also been reported: activation of the descending pain suppression pathway and direct stimulation of the thalamus by SCS ascending impulses, leading to pain relief and improvement of failed sensory modulation action in the basal ganglia [4, 13–19].

In our patient, pain relief was observed immediately after both temporary and permanent placement of the SCS device, followed by gradual improvement in postural abnormalities such as camptocormia with Pisa syndrome. This suggests indirect alleviation of postural abnormalities via pain relief by SCS, as described by Agari et al. [3]. However, the time lag between pain relief (immediately recognized after placement of the SCS device) and alleviation of postural abnormalities (gradually recognized roughly 10 days after placement of the SCS device), and the complete but transient disappearance of camptocormia after DBS, which directly affects the central nervous system (including the subthalamic nucleus or globus pallidus), suggests a pain relief-independent mechanism for the alleviation of camptocormia in our patient, in whom stimulation of the posterior funiculus resulted in improvement, directly alleviating abnormalities of central nerve circuits such as the cortico-basal ganglia-thalamic loop via the medial lemniscus-thalamic system. Such direct effects on the central and peripheral nervous systems have already been confirmed in animal models [20–22], indicating the need for studies investigating the effect of SCS in patients with camptocormia but no pain.

Notably, the scores for UPDRS part II and postural abnormalities were markedly improved in this case. Alleviation of postural abnormalities and improved motor function can be explained by an indirect effect of pain relief due to SCS, but a pain relief-independent explanation, involving direct correction of the trunk posture and consequent adjustment of the dynamic center of body mass, has also been reported [3]. Given the time difference between pain relief and alleviation of camptocormia by SCS observed in our case, neither a direct nor an indirect mechanism can be eliminated in explaining the alleviation of individual symptoms. Furthermore, improvement in activities of daily living indicates the possibility that SCS can also alleviate motor symptoms in patients with parkinsonism and motor dysfunction.

## Conclusions

This case suggests that SCS could be effective for improving camptocormia or Pisa syndrome and motor symptoms of Parkinson’s disease through pain relief, and is a treatment option for postural abnormalities such as camptocormia and Pisa syndrome and for parkinsonism with Parkinson’s-unrelated pain. There is a possibility that SCS works through a mechanism other than pain relief, and further accumulation of cases is needed.

## Abbreviations

DAT: dopamine transporter
DBS: deep brain stimulation
MRI: magnetic resonance imaging
SCS: spinal cord stimulation
UPDRS: Unified Parkinson’s Disease Rating Scale

## References

[1] Seki M, Takahashi K, Koto A, Mihara B, Morita Y, Isozumi K, Ohta K, Muramatsu K, Gotoh J, Yamaguchi K, Tomita Y, Sato H, Nihei Y, Iwasawa S, Suzuki N, on behalf of Keio Parkinson's Disease Database. Camptocormia in Japanese patients with Parkinson's disease: a multicenter study. Mov Disord. 2011;26(14):2567–71. 10.1002/mds.23955 21953897

[2] Fil A, Cano-de-la-Cuerda R, Muñoz-Hellín E, Vela L, Ramiro-González M, Fernández-de-Las-Peñas C. Pain in Parkinson disease: a review of the literature. Parkinsonism Relat Disord. 2013;19(3):285–294; discussion 285. doi:10.1016/j.parkreldis.2012.11.009. 10.1016/j.parkreldis.2012.11.009 23246139

[3] Agari T, Date I. Spinal cord stimulation for the treatment of abnormal posture and gait disorder in patients with Parkinson's disease. Neurol Med Chir (Tokyo). 2012;52:470–4. 10.2176/nmc.52.470 22850494

[4] Nishioka K, Nakajima M. Beneficial therapeutic effects of spinal cord stimulation in advanced cases of Parkinson's disease with intractable chronic pain: a case series. Neuromodulation. 2015;18:751–3. 10.1111/ner.12315 26047363

[5] De Andrade EM, Ghilardi MG, Cury RG, Barbosa ER, Fuentes R, Teixeira MJ, Fonoff ET. Spinal cord stimulation for Parkinson's disease: a systematic review. Neurosurg Rev. 2016 ;39(1):27–35; discussion 35. doi:10.1007/s10143-015-0651-1. 10.1007/s10143-015-0651-1 26219854

[6] Hughes AJ, Daniel SE, Kilford L, Lees AJ. Accuracy of clinical diagnosis of idiopathic Parkinson’s disease: a clinic-pathological study of 100 cases. J Neurol Neurosurg Psychiatry. 1992;55:181–4. 10.1136/jnnp.55.3.181 PMC1014720 1564476

[7] Doherty KM, van de Warrenburg BP, Peralta MC, Silveria-Moriyama L, Azulay J-P, Gershanik OS, Bloem BR. Postural deformities in Parkinson’s disease. Lancet Neurol. 2011;10(6):538–49. doi:10.1016/S1474-4422(11)70067-9. 10.1016/S1474-4422(11)70067-9 21514890

[8] Azher SN, Jankovic J. Camptocormia: pathogenesis, classification, and response to therapy. Neurol. 2005;65(3):355–9. 10.1212/01.wnl.0000171857.09079.9f 16087897

[9] Ho B, Prakash R, Morgan JC, Sethi KD. A case of levodopa-responsive camptocormia associated with advanced Parkinson's disease. Nat Clin Pract Neurol. 2007;3(9):526–30. 10.1038/ncpneuro0584 17805247

[10] Finsterer J, Strobl W. Presentation, etiology, diagnosis, and management of camptocormia. Eur Neurol. 2010;64(1):1–8. doi:10.1159/000314897. 10.1159/000314897 20634620

[11] Fukaya C, Otaka T, Obuchi T, Kano T, Nagaoka T, Kobayashi K, Oshima H, Yamamoto T, Katayama Y. Pallidal high-frequency deep brain stimulation for camptocormia: an experience of three cases. Acta Neurochir Suppl. 2006;99:25–8. 10.1007/978-3-211-35205-2_4 17370758

[12] Melzack R, Wall PD. Pain mechanisms: a new theory. Science. 1965;150:971–9. 10.1126/science.150.3699.971 5320816

[13] Thang TC, Janik JJ, Grill WM. Mechanisms and models of spinal cord stimulation for the treatment of neuropathic pain. Brain Res. 2014;1569:19–31. doi:10.1016/j.brainres.2014.04.039. 10.1016/j.brainres.2014.04.039 24802658

[14] Larson SJ, Sances A Jr, Riegal DH, Meyer GA, Dallmann DE, Swiontek T. Neurophysiological effects of dorsal column stimulation in man and monkey. J Neurophysiol. 1974;41:217–23. 10.3171/jns.1974.41.2.0217 4210432

[15] Campbell JN. Examination of possible mechanisms by which stimulation of the spinal cord in man relieves pain. Appl Neurophysiol. 1981;44:181–6. 10.1159/000102200 6978676

[16] Meyerson BA, Herrregodts P, Linderoth B, Ren B. An experimental animal model of spinal cord stimulation for pain. Stereotact Funct Neurosurg. 1994;62:256–62. 10.1159/000098629 7631077

[17] Duggan AW, Foong FW. Bicuculline and spinal inhibition produced by dorsal column stimulation in the cat. Pain. 1985;22:249–59. 10.1016/0304-3959(85)90025-9 2993983

[18] Stiller CO, Cui JG, O'Connor WT, Brodin E, Meyerson BA, Linderoth B. Release of GABA in the dorsal horn and suppression of tactile allodynia by spinal cord stimulation in mononeuropathic rats. Neurosurgery. 1996;39:367–75. 10.1097/00006123-199608000-00026 8832675

[19] Cui JG, O'Connor WT, Ungerstedt U, Linderoth B, Meyerson BA. Spinal cord stimulation attenuates augmented dorsal horn release of excitatory amino acids in mononeuropathy via a GABAergic mechanism. Pain. 1997;73:87–95. 10.1016/s0304-3959(97)00077-8 9414060

[20] Fuentes R, Petersson P, Nicolelis MA. Restoration of locomotive function in Parkinson's disease by spinal cord stimulation: mechanistic approach. Eur J Neurosci. 2010;32(7):1100–8. doi:10.1111/j.1460-9568.2010.07417.x. 10.1111/j.1460-9568.2010.07417.x PMC2998915 21039949

[21] Hassan S, Amer S, Alwaki A, Elborno A. A patient with Parkinson's disease benefits from spinal cord stimulation. J Clin Neurosci. 2013 Aug;20(8):1155–6. doi:10.1016/j.jocn.2012.08.018. 10.1016/j.jocn.2012.08.018 23453160

[22] Brys I, Bobela W, Schneider BL, Aebischer P, Fuentes R. Spinal cord stimulation improves forelimb use in an alpha-synuclein animal model of Parkinson's disease. Int J Neurosci. 2017;127(1):28–36. 10.3109/00207454.2016.1138296 26856727

